# Transdiagnostic factors predicting the 2-year disability outcome in patients with anxiety and depressive disorders

**DOI:** 10.1186/s12888-023-04919-1

**Published:** 2023-06-16

**Authors:** G. Margaret Ruitenberg, S. H. Sanne Booij, N. M. Neeltje Batelaan, A. W. Adriaan Hoogendoorn, H. A. Henny Visser

**Affiliations:** 1 Mental Health Care Institute GGZ Centraal, Ermelo, The Netherlands; 2 Department of Psychiatry, Interdisciplinary Center Psychopathology and Emotion Regulation, University of Groningen, University Medical Center Groningen, Huispostcode CC72, Postbus 30.001, 9700 RB Groningen, The Netherlands; 3 Center for Integrative Psychiatry, Lentis, Groningen, the Netherlands; 4 Department of Psychiatry, Amsterdam UMC, Vrije Universiteit, Amsterdam Public Health Research Institute, Amsterdam, The Netherlands; 5 Specialized Mental Health Care, GGZ InGeest, Amsterdam, The Netherlands

**Keywords:** Transdiagnostic factor, Anxiety and depressive disorders, Disability outcome, NESDA, WHODAS II, Extraversion, Locus of control, Experiential avoidance

## Abstract

**Background:**

Both anxiety and depressive disorders are associated with significant long-term disability. Since experienced impairments vary between patients independent of diagnosis and disease severity, identifying transdiagnostic factors that predict the course of disability may provide new targets to reduce disability. This study examines transdiagnostic factors predicting the 2-year disability outcome in patients with anxiety and/or depressive disorders (ADD), focusing on potentially malleable factors.

**Methods:**

Six hundred fifteen participants with a current diagnosis of ADD from the Netherlands Study of Depression and Anxiety (NESDA) were included. Disability was assessed at baseline and after 2 years of follow-up, using the 32-item WHODAS II questionnaire. Transdiagnostic predictors of 2-year disability outcome were identified using linear regression analysis.

**Results:**

In univariable analyses, transdiagnostic factors associated with the 2-year disability outcome were locus of control (standardized β = -0.116, *p* = 0.011), extraversion (standardized β = -0.123 *p* = 0.004) and experiential avoidance (standardized β = 0.139, *p* = 0.001). In multivariable analysis, extraversion had a unique predictive value (standardized β = -0.143 *p* = 0.003). A combination of sociodemographic, clinical and transdiagnostic variables resulted in an explained variance (R^2^) of 0.090). The explained variance of a combination of transdiagnostic factors was 0.050.

**Conclusion:**

The studied transdiagnostic variables explain a small but unique part of variability in the 2-year disability outcome. Extraversion is the only malleable transdiagnostic factor predictive of the course of disability independent of other variables. Due to the small contribution to the variance in the disability outcome, the clinical relevance of targeting extraversion seems limited. However, its predictive value is comparable to that of accepted disease severity measures, supporting the importance of looking beyond using disease severity measures as predictors. Furthermore, studies including extraversion in combination with other transdiagnostic and environmental factors may elucidate the unexplained part of variability of the course of disability in patients with ADD.

**Supplementary Information:**

The online version contains supplementary material available at 10.1186/s12888-023-04919-1.

## Background

Both anxiety and depressive disorders are associated with significant current and long-term disability with a similar or higher impact on daily functioning as compared to arthritis or heart disease [1–5]. Disability refers to all impairments, activity limitations or participation restrictions arising from the interaction between health conditions and contextual (i.e. environmental and personal) factors [6]. Though impairments are often regarded as a consequence of the disease, they vary substantially independent of the diagnosis and severity [6].

A transdiagnostic view on illness focuses on shared psychosocial, genetic or neurobiological factors across mental disorders in contrast to a strict disorder specific approach [7–11]. Sleep [12], emotion regulation [13, 14], alexithymia [15, 16], personality characteristics (i.e. perfectionism [17]) and cognitive patterns [18], self-esteem [19], intolerance of uncertainty [20], experiential avoidance [21], loneliness and social support [22], attachment style [23] and lifestyle [24] are examples of (presumed) transdiagnostic factors. These factors are recognized to play a role in the development and/or maintenance of symptoms associated with a broad range of psychopathology. A shift towards a transdiagnostic approach to psychopathology offers opportunities to identify shared treatment targets to improve the level of functioning. This is crucial, because the social and economic costs of disability are significant and people with disabilities have poorer health outcomes in general, lower education achievements, less economic participation and higher rates of poverty than people without disabilities [6].

The current literature is scarce and fragmented regarding transdiagnostic factors that predict the course of disability in patients with anxiety and / or depressive disorder (ADD). Hendriks et al. 2016 described the four-year disability in anxiety disorders. Higher levels of anxiety arousal and avoidance behavior, both indicators of a higher disease severity level, were major predictors for the course of disability [3]. However, transdiagnostic predictors were not considered. In a model combined with other clinical and sociodemographic variables including disease severity, Iancu et al. 2020 reported no association of personality characteristics and comorbid chronic somatic diseases with six-year residual disability in patients with remitting major depressive disorder (MDD) [4]. Persisting sleeping problems, which were specifically present among patients with a major depressive disorder or a general anxiety disorder, appeared to be associated with higher disability levels at six-month follow-up in a long-term inpatient psychiatric cohort described by Hartwig et al. 2019 [25]. Hence, to the best of our knowledge, there is a lack of studies specifically regarding potentially malleable transdiagnostic factors associated with the course of disability in patients with ADD.

Our aim is to examine which transdiagnostic factors predict the 2-year disability outcome in a mixed sample of patients with ADD, focusing on potentially malleable factors (that can be intervened on). A 2-year follow-up period, compared to 4 or 6 years, maximizes variability in the outcome [26], and is less hindered by attrition and noise due to (uncaptured) changes in time-dependent variables. Personality characteristics, multiple cognitive and behavioral patterns, lifestyle and sleep are considered as possible predictors. Specifically, to tease apart the independent and shared effects, we examined the strength of the associations in univariable and multivariable models. In addition, to assess the added value of transdiagnostic factors to often used clinical and demographic factors, we examined a model with and without these other potential predictors.

## Methods

### Design and setting

Data were obtained from the Netherlands Study of Depression and Anxiety (NESDA), a multi-site naturalistic longitudinal cohort study investigating the long-term course and consequences of ADD and their predictors looking into psychosocial, biological and genetic determinants [27]. Between September 2004 and February 2007, 2981 participants aged 18–65 years were recruited. Overall, 1701 (57%) people with a current (within the last six months) diagnosis of ADD, 907 (30%) people with life-time diagnoses or at risk of ADD and 373 (13%) healthy controls were included. Patients who were not fluent in Dutch or with a primary diagnosis of psychotic disorder, obsessive–compulsive disorder or severe addiction disorder were excluded. 564 (19%) participants were recruited from community samples, 1610 (54%) participants were recruited from primary care practices and 807 (27%) participants were recruited from mental health organizations. Trained research assistants collected the data and interviews were taped to monitor and analyze data-quality and interviewer performance. After the baseline assessment (T0), follow-up assessments took place at subsequently 2 years (T1; *N *= 2596), 4 years (T2; *N* = 2402), 6 years (T3; *N* = 2256). The research protocol was approved by the Ethical Review Boards of each participating site. Written informed consent was obtained from all participants.

### Study sample

Several transdiagnostic factors of interest were assessed at T2 of NESDA, therefore T2 was set as the baseline of this study. Drop-out from T0 to T2 was as follows: From the 1701 individuals with ADD at T0, 409 dropped out (24%). The remaining 1292 individuals consisted of 632 individuals who had an ADD (37%) at T2 and 660 individuals who were remitted (39%). In addition, out of 1280 individuals without ADD at T0, 134 individuals developed ADD at T2 (10%), which were also added to our ADD sample at T2. Of the participants with a current (6-month) diagnosis of ADD at T2 (*n* = 766), patients with data on disability at both T2 and T3 (*n* = 615) were selected.

### Diagnosis

The presence of ADD was assessed at baseline (T2) using the Composite International Diagnostic Interview (CIDI, version 2.1, Dutch), a frequently used standardized diagnostic interview with good overall reliability and validity for depressive and anxiety disorders [28, 29]. Current ADD was defined as a dysthymic disorder, major depressive disorder, social anxiety disorder, panic disorder with or without agoraphobia, agoraphobia and/or generalized anxiety disorder classified according to Diagnostic and Statistical Manual of Mental Disorders (DSM)-IV criteria in the last 6 months [30].

### Two-year disability outcome

Disability was assessed at baseline (T2) and after two years of follow-up (T3) using the World Health Organization Disability Assessment Schedule II (WHODAS II) [30]. This questionnaire measures the level of functioning and disability over the past 30 days in six domains of life: cognition (6 items, e.g., ‘in the last 30 days, how much difficulty did you have in concentrating on doing something for ten minutes?’), mobility (5 items, e.g., ‘in the last 30 days, how much difficulty did you have in standing for long periods such as 30 min?’), self-care (4 items, e.g., ‘in the last 30 days, how much difficulty did you have in washing your whole body?’), interpersonal interactions (5 items, e.g., ‘in the last 30 days, how much difficulty did you have in dealing with people you do not know?’), life activities (8 items, e.g., ‘in the last 30 days, how much difficulty did you have in your day to day work/school?’) and participation in society (8 items, e.g., ‘how much of a problem did you have in joining in community activities (for example, festivities, religious or other activities) in the same way as anyone else can?’). It consists of 36 items scored on a 5-point Likert-scale. Scores were transformed into a standard scale with a range from 0 to 100. It has a good reliability and a solid factor structure that remains consistent across different types of patient populations. With respect to validity, results are consistent with those from other measures of disability [31]. Since only 342 (55.6%) of the participants were now employed, the 32-item version of the WHODAS II (excluding work disability) was used in line with other studies, for example Iancu et al. 2020 [4, 31]. The 2-year disability outcome was defined as the disability change score over 2 years (disability T3 – disability T2).

### Baseline predictors

#### Socio-demographic data assessed at baseline (T2)

Socio-demographic factors included gender, age, country of birth, education and employment status. Level of education was divided in three subgroups: basic (elementary education not completed or elementary education), intermediate (lower/intermediate vocational education, general intermediate education or general secondary education) and high (higher vocational education, college education, university education).

#### Clinical variables assessed at baseline (T2)

##### Disease severity measures

Severity of depressive symptoms was assessed with the 30-item Inventory of Depressive Symptomatology (IDS). Items were scored on a 4-point scale (range 0–3), with a total score range from 0 to 84. The IDS has an adequate reliability, acceptable validity and a good responsiveness and discriminative ability [32–34]. Severity of overall anxiety was assessed using the 21-item Beck Anxiety Inventory (BAI) [35] and severity of avoidance symptoms was assessed using the 5-item agoraphobia and social phobia subscale of the Fear Questionnaire (FQ) [36]. Items of the BAI were scored on a 4-point scale (range 0–3), with a total score range from 0 to 63. The BAI has shown good reliability and validity [35]. Items of the FQ were scored on a 9-point scale from 0 ‘would not avoid it’ to 8 ‘always avoid it’. The total score range of both subscales is 0 to 40. The FQ has shown adequate reliability and validity [37]. Information on the number of months with depressive and anxiety symptoms in the two years prior to the baseline assessment was assessed using the Life Chart Interview [38].

##### Presence of current chronic somatic diseases under treatment

The presence of ≥ 1 current chronic somatic disease(s) under treatment by a physician or for which medication was used was assessed.

#### Potentially malleable transdiagnostic factors assessed at baseline (T2)

##### Smoking, drug use and alcohol consumption

The presence of current smoking and drug use were assessed. Current alcohol consumption was determined using the 10-item Alcohol Use Disorders Identification Test (AUDIT), the recommended cut-off score of ≥ 8 was used as an indicator of hazardous and harmful alcohol use [39]. The AUDIT has shown high reliability and acceptable validity [39].

##### Body mass index, physical activity and sleep behavior

The Body Mass Index (BMI) was assessed. The level of physical activity was measured using the 7-item International Physical Activity Questionnaire (IPAQ). It assesses the amount of vigorous, moderate, walking and sitting activities over the last seven days. The categorical score expresses three levels of physical activity: low, moderate and high. Reliability and validity of the IPAQ are both acceptable [40]. Sleep behavior was evaluated using the 5-item Women’s Health Initiative Insomnia Rating Scale (WHIIRS), the recommended cut-off score of > 8 was used to discriminate between insomnia and no insomnia [41]. Reliability and validity of the WHIIRS are both acceptable [41–43].

##### Trait approach-avoidance tendencies

Trait approach and avoidance tendencies were measured by the Behavioral Inhibition System scale (BIS) and Behavioral Activation System Drive subscale (BAS-D) [44]. The 7-item BIS scale measures behavioral inhibition sensitivity and the 4-item BAS-D scale measures behavioral activation sensitivity. Both the BIS and BAS-D have shown good reliability and validity [45].

##### Dispositional optimism

The 11-item Life Orientation Test-R (LOT-R) measures the level of dispositional optimism, with higher scores being indicative of a higher level of optimism. The LOT-R has shown acceptable reliability and adequate predictive and discriminant validity [46].

##### Cognitive reactivity to sad mood

The 34-item Leiden Index of Depression Sensitivity—Revised (LEIDS-R) measures cognitive reactivity to sad mood, with higher scores indicating stronger cognitive reactivity. The LEIDS has shown sufficient reliability and validity [47–49].

##### *Locus of control*

Locus of control (LOC) was measured using the 5-item Pearlin Mastery Scale (PMS) [50]. LOC refers to a person’s expectation that outcome is contingent on their own behavior or personal characteristics (i.e. internal) versus outcome being a function of chance, luck or fate, is under the control of others or totally unpredictable (i.e. external) [51]. A higher score indicates more feelings of mastery [50]. The PMS has shown an acceptable reliability and validity [52, 53].

##### Neuroticism and extraversion

The Dutch version of the 60-item NEO-Five Factor Inventory (NEO-FFI) measures the five most important domains of personality. [54, 55]. It In this study, the 12-item neuroticism and 12-item extraversion subscales were considered, other subscales (i.e. openness, conscientiousness, agreeableness) were not available at T2. The reliability, internal structure and construct validity of the NEO-FFI are satisfactory [55].

##### Experiential avoidance

Experiential avoidance was assessed using the 9-item Acceptance and Action Questionnaire-I [56, 57]. The internal consistency and temporal stability of the AAQ-I were satisfactory. Concerning validity, higher AAQ-I scores were associated with psychopathology and maladaptive coping strategies [57].

### Power considerations

The sample size calculation of this predictive factors research is based on an R-squared (R^2^) test in multivariable linear regression for the outcome variable ‘change in disability’. In testing a set of fourteen predictive factors by means of multivariable regression analysis using a model with ten covariates, including socio-demographic variables and baseline disability scores, that explain at least a conservative percentage of one percent of the outcome variable, assuming alpha = 0.05, a total sample size of *n* = 600 allows us to detect an effect size of at least an increase in R^2^ = 0.03 with power = 1 – beta = 0.80. This power calculation involved a set of fourteen transdiagnostic factors, assuming controlling for ten covariates that explain only a conservatively one percent of the variability and was obtained from Stata Statistical Software version 17.0 [58] using ‘power rsquared 0.01 0.04, ncontrol(10) ntested(14) n(600)’.

### Data analysis and statistical methods

Mean disability at baseline and follow-up were compared using a paired samples T-test. Strength of association between sociodemographic, clinical and transdiagnostic variables and the 2-year disability outcome was identified through univariable regression analysis (significance value set at 0.05). The dependent variable was the 2-year disability outcome defined as the disability change score over a period of 2 years corrected for baseline disability. A correction for baseline disability was applied to avoid distortions of the results due to regression to the mean.

Multicollinearity was suspected if correlation coefficients calculated prior to multivariable analysis were larger than 0.80 and if the variance inflation factor (VIF) was larger than 10 [59]. In case of a highly collinear relationship between variables, adjustment of the structure of the model and selection of independent variables was performed, to prevent a reduction of the statistical significance of the independent variables.

Independent sociodemographic, clinical and transdiagnostic predictors of the 2-year disability outcome were identified through 2 multivariable models. The significance value was set at 0.0035 after Bonferroni correction (0.05/14 transdiagnostic factors). Model 1 included all factors from univariable analysis with a p-value of < 0.25, in the spirit of purposeful variable selection [60]. To further explore the effect of transdiagnostic factors specifically, Model 2 included all transdiagnostic factors from univariable analysis with a p-value of < 0.25. The R-squared estimate indicates the model performance, with R^2^ = 1 (range 0–1) signifying a ‘perfect’ fit.

Data were analyzed using SPSS Statistics for Windows version 25.0 [61].

## Results

### Study sample

Descriptive characteristics of the study sample (*n* = 615) are summarized in Table 1. Mean age was 46.8 years (SD 12.4). Most participants were female (69.6%) and were born in the Netherlands (92.7%). Most of the participants had obtained an intermediate or high education level (93%) and more than half of the participants were currently employed (55.6%). Major depressive disorder (52.7%), social anxiety disorder (34.5%), dysthymic disorder (19.8%) and generalized anxiety disorder (18.5%) were the most common diagnoses. Anxiety disorders were most prevalent and comorbid anxiety and depressive disorder(s) were common (29.9%). Mean disability was 26.2 (SD 15.9) at baseline and 24.3 (SD 16.7) after 2 years of follow-up.

**Table 1:** Characteristics of the study population, *n* = 615

**Variables**	mean or %	SD or n	**Variables**	mean or %	SD or n
*Disability measures*			*Current diagnoses—6 month*		
Standardized WHODAS II, 32-item version (range = 0–100) T2	26.2	15.9	Dysthymic disorder	19.8%	122
Standardized WHODAS II, 32-item version (range = 0–100) T3	24.3	16.7	Major depressive disorder	52.7%	324
Standardized WHODAS II, 32-item version, change score	-1.9^a^	11.8	SociaI anxiety disorder	34.5%	212
*Socio-demographics*			Panic disorder with agoraphobia	9.4%	58
Female gender	69.6%	428	Panic disorder without agoraphobia	14.3%	88
Age	46.8	12.4	Agoraphobia	15.8%	97
Country of birth			Generalized anxiety disorder	18.5%	114
The Netherlands	92.7%	570	≥ 1 anxiety disorder(s)	71.1%	437
Other European country	3.4%	21	≥ 1 depressive disorder(s)	58.9%	362
Non-European country	3.9%	24	Cormorbid anxiety and depressive disorder(s)	29.9%	184
Education			*Presumed transdiagnostic factors*		
Basic	7.0%	43	Current smoker (*n* = 611)	31.7%	195
Intermediate	54.1%	333	Current drug use (*n* = 611)	4.2%	26
High	38.9%	239	Alcohol use Disorders Identification Test (AUDIT, range = 0–40) ≥ 8 (*n* = 614)	20.8%	128
Employment status (*n* = 601)			Women's HeaIth Initiative Insomnia Rating Scale (WHIIRS, range = 0–20) > 8 (*n* = 611)	51.5%	317
Now employed	55.6%	342	International Physical Activity Questionnaire (IPAQ, *n* = 587)		
Occupationally disabled or sickness benefit	18.4%	113	Low	26.5%	163
Retirement	7.3%	45	Moderate	39.7%	244
Not employed	16.4%	101	High	29.3%	180
*Clinical variables*			Body Mass Index (BMI, *n* = 575)	26.2	5.2
Disease severity measures			Behavioral Activation System – Drive subscale (BAS-D, range = 7–28, *n* = 614)	10.7	2.8
Inventory of Depressive Symptomatology (IDS, range = 0–84)	25.4	12.3	Behavioral Inhibition System scale (BIS, range = 4–16, *n* = 614)	12.2	3.6
Beck Anxiety Inventory (BAI, range = 0–63)	14.3	9.7	Life Orientation Test-R (LOT-R, range 0–24, *n* = 614)	11.9	3.8
Fear Questionnaire – Social phobia	14.4	8.8	Leiden Index of Depression Sensitivity—Revised (LEIDS-R, range = 0–136)	21.7	11.2
Fear Questionnaire—Agoraphobia	9.1	9.0	Pearlin Mastery Scale 5-item (PMS, range = 5–25)	15.6	4.4
Current chronic somatic diseases under treatment	46.0%	283	NEO-FFI Neuroticism (range = 5–60)	39.2	6.6
No. months with depressive symptoms (within past 2 years)	12.1	10.4	NEO-FFI Extraversion (range = 5–60)	33.8	6.7
No. months with anxiety symptoms (within past 2 years)	15.4	10.1	Acceptance & Action Questionnaire – I 9-item (AAQ-I, range = 9–63, *n* = 592)	38.6	6.9

*WHODAS II* World Health Organization Disability Assessment Schedule II, *NEO-FFI* NEO-Five Factor Inventory

^a^ Decrease in disability score (paired samples T-test, t_614_ = 4.059, *p* = 0.000)

A comparison of individuals with ADD (at T0) who did and did not drop out between T0 and T2 (this study’s baseline), is presented in Supplementary Table S1. Age, gender, education level, IDS, BAI, FQ and WHODAS-II scores were compared. In short, drop-outs were younger, had a lower education level, and had a lower score on the Fear Questionnaire – Social Phobia subscale.

### Two-year disability outcome

The mean disability change score was -1.9 (11.8), signifying a small improvement of the level of disability over a period of 2 years (t_614_ = 4.059, *p* = 0.000).

### Predictors of the 2-year disability outcome

Predictors of the 2-year disability outcome (i.e. disability change scores over a period of 2 years corrected for baseline disability) in patients with ADD are shown in Table 2. In univariable analysis, transdiagnostic factors associated with the 2-year disability outcome were LOC (β = -0.116, *p* = 0.011), extraversion (β = -0.123 *p* = 0.004) and experiential avoidance (β = 0.139, *p* = 0.001).

**Table 2:** .

	**Univariable**					**Multivariable**^**c**^				
			M1^I^ *R*^*2*^ = *0.090*^*d*^				M2^II^ *R*^*2*^ = *0.050*^*d*^			
	β^b^	*p*-value	β^b^	B	95%CI	*p*-value	β^b^	B	95%CI	*p*-value
**Sociodemographics**										
** Female gender**	-0.021	0.588								
** Age**	0.091	**0.019**	0.032	0.030	-0.058 – 0.118	0.666				
**Country of birth**										
** The Netherlands**	ref	ref								
** Other European country**	0.028	0.474								
** Non-European country**	0.098	**0.011**								
**Education**										
**Basic**	ref	ref								
** Intermediate**	-0.126	**0.102**	-0.086	-1.972	-6.000 – 2.055	0.336				
** High**	-0.220	**0.005**	-0.129	-2.996	-7.207 – 1.215	0.163				
**Employment status—paid job**	0.071	**0.084**	0.022	0.496	-1.531 – 2.523	0.631				
**Clinical variables**										
** IDS**	0.140	**0.025**	0.022	0.021	-0.131 – 0.173	0.783				
** BAI**	0.153	**0.002**	0.082	0.099	-0.044 – 0.241	0.175				
** FQ—Social phobia**	0.138	**0.002**	0.063	0.082	0.069 – 0.233	0.289				
** FQ—Agoraphobia**	0.160	** < 0.001**	0.052	0.066	-0.074 – 0.207	0.355				
** No. months depressive symptoms**	0.066	**0.110**	0.022	0.024	-0.080 – 0.128	0.647				
** No. months anxiety symptoms**	0.074	**0.057**	0.011	0.013	-0.084 – 0.109	0.796				
** CCSDUT**	0.113	**0.004**	0.078	1.784	-0.338 – 3.907	0.099				
**Transdiagnostic factors**			*R*^*2*^ = *0.032*^*e*^							
**Current smoker**	0.027	0.488								
**Current drug use**	0.049	**0.209**	0.056	3.144	-1.556 – 7.843	0.189	0.051	2.915	-1.742 – 7.573	0.219
**AUDIT ≥ 8**	-0.004	0.916								
**WHIIRS > 8**	0.068	**0.085**	0.029	0.669	-1.305 – 2.544	0.506	0.039	0.897	-1.011 – 2.805	0.356
**IPAQ**										
** Low**	ref	ref	ref			ref	ref			ref
** Moderate**	-0.068	**0.165**	-0.080	-1.857	-3.798 – 0.085	0.061	-0.073	-1.683	-3.580 – 0.216	0.082
** High**	0.000	0.994								
**BMI**	0.065	**0.114**	0.041	0.089	-0.103 – 0.280	0.363	0.067	0.146	-0.037 – 0.328	0.118
**BAS-D**	-0.010	0.791								
**BIS**	0.035	0.367								
**LOT-R**	-0.065	**0.129**	0.038	0.114	-0.213 – 0.440	0.493	0.024	0.072	-0.250 – 0.393	0.661
**LEIDS-R**	0.067	**0.140**	0.034	0.036	-0.071 – 0.144	0.510	0.050	0.052	-0.052 – 0.157	0.328
**PMS**	-0.116	**0.011**	-0.052	-0.137	-0.439 – 0.165	0.374	-0.055	-0.146	-0.442 – 0.150	0.332
**NEO-FFI Neuroticism**	0.030	0.496								
**NEO-FFI Extraversion**	-0.123	**0.004**	-0.146	-0.256	-0.440 – -0.072	0.007	-0.143	-0.252	-0.419 – -0.086	**0.003**
**AAQ-I**	0.139	**0.001**	0.061	0.102	-0.070 – 0.273	0.245	0.075	0.125	-0.040 – 0.290	0.137

*ADD* anxiety and/or depressive disorder, *IDS* Inventory of Depressive Symptomatology, *BAI* Beck Anxiety Inventory, *CCSDUT* current chronic somatic diseases under treatment, *AUDIT* Alcohol Use Disorders Identification Test, *WHIIRS* Women's Health Initiative Insomnia Rating Scale, *IPAQ* International Physical Activity Questionnaire, *BMI* Body Mass Index, *BAS-D* Behavioral Activation System—Drive subscale, *BIS* Behavioral Inhibition System scale, *LOT-R* Life Orientation Test—Revised, *LEIDS-R* Leiden Index of Depression Sensitivity—Revised

*PMS* Pearlin Mastery Scale, *AAQ-I* Acceptance & Action Questionnaire—I

^a^ Disability change score over 2 years (disability T3 – disability T2) corrected for baseline disability measured using the 32-item World Health Organization Disability Assessment Schedule II (WHODAS II). Improvement (i.e. a decrease in disability severity level) is recognized by a negative change score and a negative β

^b^ standardized β coefficient

^c^ significant factors retrieved from univariate analysis were excluded from the consecutive multivariable models if present (i.e. scored positive) in ≤ 50 people (8.1%) of the total sample; a non-European birth country was excluded

^d^ Predictive value (R-squared) of Model 1 and 2

^e^ Predictive value (R-squared) of the transdiagnostic factors in Model 1

^I^ Model 1: purposefully selected variables from univariate analysis, *p*-value < 0.25, ^II^ Model 2: purposefully selected transdiagnostic factors from univariate analysis, *p*-value < 0.25

The VIF of the independent variables varied between 1.0 and 5.0 and the largest correlation coefficient was 0.79 (Supplementary Table S2). Multicollinearity was not a problem.

In multivariable analysis of purposefully selected sociodemographic, clinical and transdiagnostic variables (Model 1), the total explained variance (R^2^, excluding explained variance by baseline disability) was 0.090. The explained variance of the transdiagnostic factors in Model 1 was 0.032. Among the transdiagnostic factors, extraversion was predictive of the 2-year disability outcome (β = -0.173 *p* = 0.001). In multivariable analysis of purposefully selected transdiagnostic variables (Model 2), the explained variance of the transdiagnostic variables was 0.050.

## Discussion

### Findings

Our aim was to examine transdiagnostic factors predictive of the 2-year disability outcome in patients with ADD, focusing on potentially malleable factors. The mean disability change score was negative, signifying a small improvement of the level of disability over a period of 2 years. Unfortunately, there are no established thresholds for interpreting the global or domain-specific scores in relation to the criterion of clinically significant impairment.

The first multivariable model considering a combination of purposefully selected sociodemographic, clinical and transdiagnostic factors (i.e. Model 1) explained 9% of the total variability in the 2-year disability outcome. Remarkably, the total variance explained by the model is about five times lower than reported by Iancu et al. 2020 describing comparable predictors of the six-year residual disability in patients with remitting MDD [4]. This is partly explained by their choice to include baseline disability as a predictor instead of performing a statistical correction. Another possible explanation is their more restricted study sample; the differences in results might indicate that there is a lack of overlap between predictors of the course of disability in anxiety and depressive disorders. However, ter Meulen et al. 2021 reported > 75% comorbidity in subjects with ADD. Transitions between depressive and anxiety disorders were common [62]. The beforementioned simultaneous and alternating occurrence of ADD indicate that anxiety and depressive disorders are less distinct entities than suggested by current classification systems and that they are likely to share predictors of the course of the disease. Other possible explanations for the difference in results are a stronger association of the predictors with more long-term disability.

Our study complements earlier research by providing evidence regarding the association of transdiagnostic factors with the course of disability. Results of univariable analysis showed that a higher degree of LOC, a higher level of extraversion and a lower level of experiential avoidance were transdiagnostic factors predictive of a more favorable 2-year disability outcome.

In multivariable analysis, the additional amount of variance explained by transdiagnostic variables was small to medium, both apart from and in combination with sociodemographic and clinical variables. These findings indicate that the examined transdiagnostic factors explain a small but unique part of the degree of variability in the 2-year disability outcome.

In both multivariable analyses (i.e. Model 1 and 2), a lower level of extraversion was predictive of an unfavorable 2-year disability outcome. Personality traits were originally believed to be stable between individuals over time, and changes over the life course were thought to follow common patterns induced primarily by brain maturation and genetic factors [63]. However, together with cumulating evidence, newer theories, such as the Experience-dependent and Mixed set-point model, emphasize that both genetic influences as well as cognitive and environmental influences can drive long-lasting changes [63]. In line with this theory, in their literature review, Roberts et al. 2017 describe evidence that the level of extraversion can change because of intervention [64]. Consequently, targeting extraversion may improve the 2-year disability outcome, but due to the small contribution to the variance in the disability outcome, the clinical relevance of targeting extraversion seems limited. Yet, it is noteworthy that extraversion has a comparable strength of association with the 2-year disability outcome as compared to the accepted and widely used disease severity measures (i.e. IDS and BAI), supporting the importance of looking beyond using disease severity measures as predictors.

### Strengths and limitations

This study has a few limitations. First, the assessment of several variables relies on self-report questionnaires. This is important to obtain the patient’s perspective, but results might be affected by information-processing bias. Although the 32-item version of the WHODAS II was used, this does not hinder comparison to other studies, because the scores of the 32-item and 36-item WHODAS II are comparable [30]. Secondly, sufficient data on the course and type of psychotherapy and psychopharmacotherapy and the level of pre-morbid functioning were not available and therefore, their impact on the results could not be determined. Also, other potential transdiagnostic factors that were not assessed in this study, such as other personality characteristics, emotion regulation, alexithymia, self-esteem, intolerance of uncertainty, loneliness, social support and attachment style might turn out to predict the yet unexplained part of the variability. And besides personal factors, environmental factors may account for a part of the variability as well [6].

Thirdly, setting the baseline at T2 of NESDA could have led to bias, due to selective loss-to-follow up between T0 and T2. However, this is not likely as Lamers et al. 2012 found that the overall attrition over a period of 2 years was very limited [65]. Fourthly, due to the observational nature of this study, no definitive conclusions regarding causality can be substantiated.

Strengths of this study are the prospective design and the inclusion of a large mixed sample recruited from different settings, resulting in a higher degree of external validity. Another strength is the use of a rich dataset with multiple putative transdiagnostic predictors.

## Conclusion

Despite examining a combination of multiple sociodemographic, clinical and transdiagnostic predictors over a period of 2 years, most of the variability in the disability outcome in patients with ADD remains unexplained. The studied transdiagnostic variables explain a small but unique part of variability in the 2-year disability outcome. Extraversion is the only malleable transdiagnostic factor predictive of the course of disability independent of other variables. Due to the small contribution to the variance in the disability outcome, the clinical relevance of targeting extraversion seems limited. However, its predictive value is comparable to that of accepted disease severity measures, supporting the importance of looking beyond using disease severity measures as predictors. Furthermore, studies including extraversion in combination with other transdiagnostic and environmental factors may elucidate the unexplained part of variability of the course of disability in patients with ADD.

## Supplementary Information

**Additional file 1.** 

**Additional file 2.** 

## Data Availability

The data that support the findings of this study are available from the Netherlands Study of Depression and Anxiety (NESDA). Restrictions apply to the availability of these data, which were used under license for this study. Data are available www.nesda.nl with the permission of NESDA.

## Abbreviations

ADD: Anxiety and depressive disorders
NESDA: Netherlands Study of Depression and Anxiety
MDD: Major depressive disorder
CIDI: Composite International Diagnostic Interview
DSM: Diagnostic and Statistical Manual of Mental Disorders
WHODAS II: World Health Organization Disability Assessment Schedule II
IDS: Inventory of Depressive
BAI: Beck Anxiety Inventory
AUDIT: Alcohol Use Disorders Identification Test
BMI: Body Mass Index
IPAQ: Physical Activity
WHIIRS: Women’s Health Initiative Insomnia Rating Scale
BIS: Behavioral Inhibition System scale
BAS-D: Behavioral Activation System Drive subscale
LOT-R: Life Orientation Test-R
LEIDS-R: Leiden Index of Depression Sensitivity—Revised
LOC: Locus of control
PMS: Pearlin Mastery Scale
NEO-FFI: NEO-Five Factor Inventory
AAQ-I: Acceptance and Action Questionnaire-I
R^2^: R-squared
VIF: Variance inflation factor
