# Supplementary material for: Transdiagnostic factors predicting the 2-year disability outcome in patients with anxiety and depressive disorders
Source: BMC Psychiatry. 2023 Jun 16;23:443. doi: 10.1186/s12888-023-04919-1 (PMC10273546; doi:10.1186/s12888-023-04919-1)
Supplement: Supplementary file 1 — Additional file 1. [file 12888_2023_4919_MOESM1_ESM.docx]

Supplementary Table S1. Baseline comparisons of subjects with ADD who completed wave T2 and those who dropped out.

| **Baseline characteristics** | **Completed T2** | **Dropped out T2** | ***P-value**** |
| --- | --- | --- | --- |
| *N* | 632 | 409 |  |
| Age in years, mean (SD) | 42.3 (12.2) | 40.3 (12.5) | 0.01 |
| Gender, % female | 68.0 | 68.0 | 0.98 |
| Education level, % basic | 8.2 | 13.9 | <0.01 |
| Education level, % intermediate | 60.6 | 62.1 |  |
| Education level, % high | 31.2 | 24.0 |  |
| Inventory of Depressive Symptoms | 31.4 (12.9) | 30.2 (13.8) | 0.17 |
| Beck Anxiety Inventory, mean (SD) | 18.7 (10.8) | 18.7 (12.7) | 0.94 |
| Fear Questionnaire Social phobia, mean (SD) | 16.6 (8.9) | 15.0 (9.9) | 0.01 |
| Fear Questionnaire Agoraphobia, mean (SD) | 11.5 (10.0) | 10.6 (10.8) | 0.18 |
| Disability (WHODAS-II, standardized total  Score 32-item, mean (SD) | 37.4 (17.4) | 35.6 (18.5) | 0.11 |

*T-test for continuous variables and Chi-square for categorical variables.
