# Supplementary material for: Transdiagnostic factors predicting the 2-year disability outcome in patients with anxiety and depressive disorders
Source: BMC Psychiatry. 2023 Jun 16;23:443. doi: 10.1186/s12888-023-04919-1 (PMC10273546; doi:10.1186/s12888-023-04919-1)
Supplement: Supplementary file 2 — Additional file 2. [file 12888_2023_4919_MOESM2_ESM.docx]

Table S2. Correlation matrix

|  | | | | | Female gender | Age | CoB The Netherlands | CoB Other European country | CoB Non-European country | Education | Paid job | Inventory of Depressive Symptomatology (IDS) | Beck Anxiety Inventory (BAI) | Fear Questionnaire Social phobia (FQ-S) | Fear Questionnaire Agoraphobia (FQ-A) | No. Months depress symptoms | No. Months anxiety symptoms | Current smoker | Current drug use | Alcohol Use Disorders Identification Test (AUDIT) ≥8 | Women's Health Initiative Insomnia Rating Scale (WHIIRS) >8 | Current chronic somatic diseases  under treatment | International Physical Activity  Questionnaire (IPAQ) | Body Mass Index (BMI) | Behavioral Activation System - Drive subscale (BAS-D) | Behavioral Inhibition System scale (BIS) | Life Orientation Test - R (LOT-R) | Leiden Index of Depression Sensitivity - Revised (LEIDS-R) | Pearlin Mastery Scale 5-item | NEO-FFI Neuroticism | | NEO-FFI Extraversion | Acceptance & Action  Questionnaire - I 9-item (AAQ-I) | Childhood Trauma Questionnaire -  Short Form 24-item (CTQ-SF) | Standardized WHODAS II  32-item version T2 | Standardized WHODAS II  32-item version T3 | WHODAS II 32-item version change score |
| --- | --- | --- | --- | --- | --- | --- | --- | --- | --- | --- | --- | --- | --- | --- | --- | --- | --- | --- | --- | --- | --- | --- | --- | --- | --- | --- | --- | --- | --- | --- | --- | --- | --- | --- | --- | --- | --- |
| Female gender | | | | | 1 |  |  |  |  |  |  |  |  |  |  |  |  |  |  |  |  |  |  |  |  |  |  |  |  |  | |  |  |  |  |  |  |
| Age | | | | | -0.060 | 1 |  |  |  |  |  |  |  |  |  |  |  |  |  |  |  |  |  |  |  |  |  |  |  |  | |  |  |  |  |  |  |
| CoB The Netherlands | | | | | -0.050 | -0.009 | 1 |  |  |  |  |  |  |  |  |  |  |  |  |  |  |  |  |  |  |  |  |  |  |  | |  |  |  |  |  |  |
| CoB Other European country | | | | | 0.008 | -0.031 | - | 1 |  |  |  |  |  |  |  |  |  |  |  |  |  |  |  |  |  |  |  |  |  |  | |  |  |  |  |  |  |
| CoB Non-European country | | | | | 0.060 | 0.041 | - | - | 1 |  |  |  |  |  |  |  |  |  |  |  |  |  |  |  |  |  |  |  |  |  | |  |  |  |  |  |  |
| Education | | | | | 0.078 | -,140** | 0.091 | 0.095 | 0.033 | 1 |  |  |  |  |  |  |  |  |  |  |  |  |  |  |  |  |  |  |  |  | |  |  |  |  |  |  |
| Paid job | | | | | 0.009 | ,281** | 0.007 | -0.001 | -0.009 | 0,199^^ | 1 |  |  |  |  |  |  |  |  |  |  |  |  |  |  |  |  |  |  |  | |  |  |  |  |  |  |
| Inventory of Depressive Symptomatology (IDS) | | | | | -,145** | ,138** | -,083* | 0.059 | 0.057 | -,225** | ,295** | 1 |  |  |  |  |  |  |  |  |  |  |  |  |  |  |  |  |  |  | |  |  |  |  |  |  |
| Beck Anxiety Inventory (BAI) | | | | | -0.033 | 0.062 | 0.014 | -0.035 | 0.014 | -,232** | ,201** | ,673** | 1 |  |  |  |  |  |  |  |  |  |  |  |  |  |  |  |  |  | |  |  |  |  |  |  |
| Fear Questionnaire Social phobia (FQ-S) | | | | | -0.052 | 0.019 | 0.016 | -0.031 | 0.007 | -,117** | ,101* | ,383** | ,305** | 1 |  |  |  |  |  |  |  |  |  |  |  |  |  |  |  |  | |  |  |  |  |  |  |
| Fear Questionnaire Agoraphobia (FQ-A) | | | | | 0.016 | ,096* | 0.011 | -0.010 | -0.006 | -,226** | ,179** | ,406** | ,472** | ,577** | 1 |  |  |  |  |  |  |  |  |  |  |  |  |  |  |  | |  |  |  |  |  |  |
| No. months depress symptoms | | | | | -0.076 | 0.097* | -0.042 | 0.077 | -0.016 | -0.106** | 0.150** | 0.425** | 0.210** | 0.097 | 0.100* | 1 |  |  |  |  |  |  |  |  |  |  |  |  |  |  | |  |  |  |  |  |  |
| No. months anxiety symptoms | | | | | -0.082* | -0.015 | 0.016 | 0.000 | -0.021 | -0.138** | 0.079 | 0.107** | 0.193** | 0.170** | 0.227** | 0.140** | 1 |  |  |  |  |  |  |  |  |  |  |  |  |  | |  |  |  |  |  |  |
| Current smoker | | | | | -0.038 | -0.029 | 0.018 | 0.025 | -0.048 | 0,107^ | 0,089^ | 0.050 | 0.072 | -0.016 | ,107** | 0.060 | -0.005 | 1 |  |  |  |  |  |  |  |  |  |  |  |  | |  |  |  |  |  |  |
| Current drug use | | | | | -0.054 | -,178** | 0.028 | 0.005 | -0.043 | 0.019 | -0.004 | -0.047 | -0.041 | -0.002 | -0.048 | 0.002 | -0.065 | 0,203^^ | 1 |  |  |  |  |  |  |  |  |  |  |  | |  |  |  |  |  |  |
| Alcohol Use Disorders Identification Test (AUDIT) ≥8 | | | | | -0,253^^ | 0.028 | 0.021 | 0.014 | -0.041 | 0.085 | -0.053 | 0.072 | 0.052 | 0.062 | 0.019 | 0.070 | 0.034 | 0,133^^ | 0,150^^ | 1 |  |  |  |  |  |  |  |  |  |  | |  |  |  |  |  |  |
| Women's Health Initiative Insomnia Rating Scale (WHIIRS) >8 | | | | | 0.004 | ,189** | -0.002 | 0.020 | -0.016 | 0.084 | 0.059 | ,249** | ,189** | ,090* | 0.071 | 0.136** | 0.061 | -0.078 | -0,089^ | 0.022 | 1 |  |  |  |  |  |  |  |  |  | |  |  |  |  |  |  |
| Current chronic somatic diseases  under treatment | | | | | 0.007 | ,384** | -0.016 | -0.030 | 0.050 | 0,134^^ | 0,187^^ | ,164** | ,167** | 0.026 | ,098* | 0.045 | -0.016 | -0.052 | -0.064 | -0.079 | 0,146^^ | 1 |  |  |  |  |  |  |  |  | |  |  |  |  |  |  |
| International Physical Activity  Questionnaire (IPAQ) | | | | | 0.061 | -,106* | 0.081 | 0.071 | 0.044 | 0,138^^ | 0,154^^ | -,229** | -,156** | -0.054 | -,107** | -0.071 | -0.121** | 0,112^ | 0.042 | 0.053 | 0.049 | 0.012 | 1 |  |  |  |  |  |  |  | |  |  |  |  |  |  |
| Body Mass Index (BMI) | | | | | -0.080 | ,149** | 0.037 | -0.013 | -0.036 | -,256** | ,117** | ,227** | ,194** | 0.066 | ,125** | 0.146** | 0.100* | -0.026 | -,093* | -0.038 | ,097* | ,263** | -,111** | 1 |  |  |  |  |  |  | |  |  |  |  |  |  |
| Behavioral Activation System - Drive subscale (BAS-D) | | | | | -0.034 | ,130** | -0.006 | 0.014 | -0.005 | 0.023 | 0.018 | 0.033 | -0.024 | ,092* | 0.028 | 0.001 | 0.000 | -0.036 | -0.047 | -0.053 | -0.030 | 0.029 | -0.015 | 0.025 | 1 |  |  |  |  |  | |  |  |  |  |  |  |
| Behavioral Inhibition System scale (BIS) | | | | | -0.060 | ,142** | -0.040 | -0.003 | 0.057 | -,085* | 0.069 | -,159** | -,124** | -,393** | -,140** | -0.044 | -0.090* | 0.067 | 0.029 | 0.012 | -0.058 | 0.049 | -0.060 | 0.034 | -0.056 | 1 |  |  |  |  | |  |  |  |  |  |  |
| Life Orientation Test - R (LOT-R) | | | | | ,144** | -,086* | 0.036 | -0.040 | -0.011 | ,143** | -,152** | -,528** | -,364** | -,329** | -,266** | -0.268** | -0.237** | -0.067 | 0.039 | -0.077 | -,121** | -,087* | ,132** | -,178** | -,089* | ,221** | 1 |  |  |  | |  |  |  |  |  |  |
| Leiden Index of Depression Sensitivity - Revised (LEIDS-R) | | | | | -,109** | -0.049 | -0.017 | -0.016 | 0.038 | -,089* | ,139** | ,528** | ,405** | ,324** | ,214** | 0.392** | 0.067 | 0.052 | 0.007 | ,080* | ,094* | 0.025 | -0.048 | ,125** | -,097* | -,243** | -,399** | 1 |  |  | |  |  |  |  |  |  |
| Pearlin Mastery Scale 5-item (PMS) | | | | | ,086* | -,127** | 0.028 | -0.033 | -0.006 | ,212** | -,170** | -,572** | -,379** | -,375** | -,340** | -0.313** | -0.139** | -0.057 | 0.044 | -0.059 | -,158** | -,091* | ,163** | -,150** | -0.059 | ,227** | ,578** | -,483** | 1 |  | |  |  |  |  |  |  |
| NEO-FFI Neuroticism | | | | | -0.052 | -0.037 | 0.048 | 0.007 | -0.072 | -0.076 | 0.064 | ,519** | ,400** | ,494** | ,306** | 0.211** | -.130** | -0.020 | 0.013 | 0.037 | ,135** | -0.062 | -,087* | 0.080 | ,139** | -,470** | -,567** | ,534** | -,600** | 1 | |  |  |  |  |  |  |
| NEO-FFI Extraversion | | | | | ,235** | -,183** | 0.001 | -0.001 | 0.000 | 0.042 | -,188** | -,439** | -,207** | -,468** | -,253** | -0.268** | -0.064 | -0.065 | 0.005 | -0.045 | -0.042 | -0.051 | ,147** | -0.049 | -,209** | ,189** | ,430** | -,294** | ,373** | -,434** | | 1 |  |  |  |  |  |
| Acceptance & Action  Questionnaire - I 9-item (AAQ-I) | | | | | -,142** | 0.017 | -0.074 | 0.050 | 0.053 | -,232** | ,118** | ,417** | ,340** | ,359** | ,264** | 0.159** | 0.152** | 0.060 | -0.029 | 0.012 | ,094* | 0.057 | -,148** | ,136** | 0.046 | -,311** | -,481** | ,417** | -,450** | ,547** | | -,325** | 1 |  |  |  |  |
| Childhood Trauma Questionnaire - Short Form 24-item (CTQ-SF) | | | | | ,092* | ,112** | -0.059 | -0.023 | ,101* | -,166** | ,149** | ,219** | ,157** | ,197** | ,167** | 0.248** | 0.011 | ,092* | 0.033 | -0.003 | ,124** | ,106** | -,097* | ,141** | 0.045 | 0.039 | -,192** | ,224** | -,214** | ,169** | | -,174** | ,174** | 1 |  |  |  |
| Standardized WHODAS II  32-item version T2 | | | | | -0.041 | ,128** | -0.052 | 0.008 | 0.063 | -,215** | ,308** | ,785** | ,605** | ,483** | ,498** | 0.355** | 0.111* | 0.056 | -0.064 | 0.047 | ,159** | ,203** | -,244** | ,200** | 0.014 | -,131** | -,437** | ,523** | -,541** | ,464** | | -,426** | ,355** | ,283** | 1 |  |  |
| Standardized WHODAS II  32-item version T3 | | | | | -0.045 | ,158** | -,101* | 0.022 | ,114** | -,243** | ,274** | ,619** | ,516** | ,432** | ,453** | -0.281** | -0.149** | 0.060 | -0.013 | 0.032 | ,165** | ,227** | -,161** | ,191** | 0.003 | -0.072 | -,360** | ,421** | -,458** | ,360** | | -,387** | ,348** | ,244** | ,740** | 1 |  |
| WHODAS II 32-item version change score | | | | | -0.009 | 0.052 | -0.073 | 0.021 | 0.078 | -0.053 | -0.029 | -,182** | -,084* | -0.039 | -0.029 | -0.062 | 0.035 | 0.010 | 0.067 | -0.018 | 0.019 | 0.048 | ,084* | 0.005 | -0.014 | 0.074 | 0.079 | -,108** | ,080* | -,116** | | 0.027 | 0.019 | -0.036 | -,299** | ,421** | 1 |
|  |  |  |  | ** correlation is significant at the 0.01 level (2-tailed); * correlation is significant at the 0.05 level (2-tailed); ^^ correlation is significant at the 0.01 level; ^ correlation is significant at the 0.05 level | | | | | | | | | | | | | | | | | | | | | | | | | | |  |  |  |  |  |  |  |
|  |  |  |  | CoB = country of birth, IDS = Inventory of Depressive Symptomatology, BAI = Beck Anxiety Inventory, CCSDUT = current chronic somatic diseases under treatment, AUDIT = Alcohol Use Disorders Identification Test, WHIIRS = Women's Health Initiative Insomnia Rating Scale, | | | | | | | | | | | | | | | | | | | | | | | | | | |  |  |  |  |  |  |  |
|  |  |  |  | IPAQ = International Physical Activity Questionnaire, BMI = Body Mass Index, BAS-D= Behavioral Activation System - Drive subscale, BIS = Behavioral Inhibition System scale, LOT-R = Life Orientation Test - Revised, LEIDS-R = Leiden Index of Depression Sensitivity - Revised | | | | | | | | | | | | | | | | | | | | | | | | | | |  |  |  |  |  |  |  |
|  |  |  |  | PMS = Pearlin Mastery Scale, AAQ-I = Acceptance & Action Questionnaire - I, WHODAS II-32 = Standardized WHODAS II 32-item version   \| a Pearson correlation, r \| continuous versus continuous variable \| c Spearman's correlation, rs \| ordinal versus continuous variable \| e Cramér's V \| categorial versus categorial/dichotomous variable \| \| --- \| --- \| --- \| --- \| --- \| --- \| \| b Point-biserial correlation, r_pb_ \| dichotomous versus continuous variable \| d Phi \| dichotomous versus dichotomous variable \| | | | | | | | | | | | | | | | | | | | | | | | | | | |  |  |  |  |  |  |  |
